# Quality control for Illumina 450K methylation data in the absence of iDat files using correlation structure in pedigrees and repeated measures

**DOI:** 10.1186/s12863-018-0636-5

**Published:** 2018-09-17

**Authors:** Marissa LeBlanc, Haakon E. Nustad, Manuela Zucknick, Christian M. Page

**Affiliations:** 1 Oslo Centre for Biostatistics and Epidemiology, Oslo University Hospital, Klaus Torgårds vei 3, 0372 Oslo, Norway; 2 Department of Medical Genetics, Oslo University Hospital, Kirkeveien 166, 0450 Oslo, Norway; 3 Faculty of Medicine, University of Oslo, Klaus Torgårds vei 3, 0372 Oslo, Norway; 4 PharmaTox Strategic Research Initiative, University of Oslo, Oslo, Norway; 5 Oslo Centre for Biostatistics and Epidemiology, University of Oslo, Sognsvannsveien 9, 0372 Oslo, Norway; 6 Department of Non-Communicable Disease, Norwegian Institute of Public Health, Marcus Thranes Gate, Marcus Thranes gate 6, 0473 Oslo, Norway

**Keywords:** Data integration, Mediation analysis, Indirect effect

## Abstract

**Background:**

An important feature in many genomic studies is quality control and normalization. This is particularly important when analyzing epigenetic data, where the process of obtaining measurements can be bias prone. The GAW20 data was from the Genetics of Lipid Lowering Drugs and Diet Network (GOLDN), a study with multigeneration families, where DNA cytosine-phosphate-guanine (CpG) methylation was measured pre- and posttreatment with fenofibrate. We performed quality control assessment of the GAW20 DNA methylation data, including normalization, assessment of batch effects and detection of sample swaps.

**Results:**

We show that even after normalization, the GOLDN methylation data has systematic differences pre- and posttreatment. Through investigation of (a) CpGs sites containing a single nucleotide polymorphism, (b) the stability of breeding values for methylation across time points, and (c) autosomal gender-associated CpGs, 13 sample swaps were detected, 11 of which were posttreatment.

**Conclusions:**

This paper demonstrates several ways to perform quality control of methylation data in the absence of raw data files and highlights the importance of normalization and quality control of the GAW20 methylation data from the GOLDN study.

## Background

Genome-wide DNA methylation (DNAm) studies, particularly those using chip-based technologies, are inherently more susceptible to biases than single nucleotide polymorphism (SNP) studies. To make DNAm detectable, DNA is treated with bisulfite to deaminate unmethylated cytosine to uracil. Bisulfite conversion run in separate batches can lead to technical bias in DNAm studies. Bisulfite conversion can either (a) underconvert the unmethylated cytosines or (b) if not properly controlled, cause methylated cytosines to convert to uracil.

The Illumina Infinium Human Methylation 450K BeadChip (450K Chip) has two different probe chemistries (Type I and Type II) [1], which creates problems with respect to normalization. Multiple R packages for normalization and quality control (QC) for the 450K Chip have been developed [2–5]. However, most require raw data (iDat files) to run and these were not made available to GAW20 participants.

Type I probes are mainly confined to cytosine-phosphate-guanine (CpG)-rich regions; CpG richness is also associated with biological function [6]. Type I probes have two beads on the same color channel, one for methylated signal and the second for unmethylated signal. Conversely, Type II probes only have one bead that is detectable on two color channels, with one color for methylated and one for unmethylated signal. The two probe types differ in terms of dynamic range and result in different distributions for DNAm. Methods for normalizing for probe type include subset-quantile within array normalization (SWAN) [7], funNorm [8], and beta-mixture quantile normalization (BMIQ) [9]. The BMIQ method does not require raw data.

The GAW20 real data set is from the Genetics of Lipid Lowering Drugs and Diet Network (GOLDN) [10], where DNAm was measured using the 450K Chip in multigenerational pedigrees, pre- and posttreatment with a lipid-lowering drug (fenofibrate). DNAm data was provided to GAW20 participants in processed text format, after undergoing some normalization procedures [11]. However, some outstanding normalization and QC issues with the GOLDN DNAm data remain.

We address normalization and QC of the GAW20 GOLDN DNAm data. We utilize repeated DNAm measures, SNP genotype data, and family structure, and show how these can be used to detect possible sample swaps and enhance the QC process.

## Methods

### Data

All individuals in GOLDN having DNAm measurements (assayed with the 450K Chip) either pre- or posttreatment were included in the analysis (*n*_pre_ = 993, *n*_post_ = 528, *n*_both_ = 444 after removal of a twin pair) [10–12]. In addition to DNAm, age, gender, and SNP genotypes were used. SNP genotypes (Affymetrix HumanSNP array 6.0) were available for 427 of the *n*_both_ = 444 individuals. For GAW20, only autosomal DNAm was available.

### Probe-type normalization

We first visualized the DNAm data using principal component analysis (PCA) to look for systematic patterns, and then normalized the data for probe type using beta-mixture quantile normalization (BMIQ) with the R {wateRmelon} package [9]. For each individual (separately for pre- and posttreatment), BMIQ fits a three-group beta distribution mixture model separately to the Type I and Type II probes. The resulting distributions are then used to map the Type II probes to the Type I probe distribution. For all subsequent analysis, we use these DNAm beta values, unless otherwise indicated.

### Detection of potential sample swaps

We used several approaches for detecting potential sample swaps:

1. *SNPs located CpG sites:* A number of probes on the 450K Chip detect CpGs which are polymorphic and thus show genotype-specific methylation. We used 597 of these CpGs as a tool to check for sample swap by comparing CpG-inferred genotypes pre- and posttreatment. For 59 of the 597 CpGs, we were able to compare the CpG-inferred genotypes to SNP genotypes from the Affymetrix array.
2. *CpGs associated with gender:* Because the GAW20 DNAm data did not include sex chromosomes, we could not look for gender-discordant samples directly. Based on a previous study reporting gender-associated CpGs [13], we used PCA with the top 50 autosomal gender-associated CpGs to detect discordant genders compared to reported gender.
3. *Individual breeding values:* A Bayesian mixed-effects model was fitted for each CpG site separately to estimate individual breeding values, modeled as the realizations of a random intercept [14]. For inference, integrated nested Laplace approximation (INLA) [15, 16] was used (for information on INLA, see The R-INLA Project [16]). The model was constructed separately for pre- and posttreatment methylation:

$$ {y}_i\mid {\eta}_i,{\sigma}_e^2\sim N\left({\eta}_i,{\sigma}_e^2\right)\forall i=1,\dots, n\ \mathrm{with}\ {\eta}_i={\beta}_0+{\beta}_1{\mathrm{age}}_i+{\beta}_2{\mathrm{gender}}_i+{u}_i $$

$$ \boldsymbol{u}\mid {\sigma}_g^2\sim N\left(\mathbf{0},2{\sigma}_g^2\boldsymbol{K}\right)\ \mathrm{with}\ \boldsymbol{u}={\left({u}_1,\dots, {u}_n\right)}^T $$

where *y*_*i*_ is DNAm [M value: log_2_(beta/1-beta)] for person *i* at a CpG site; ${\sigma}_e^2$ is the environmental variance, *β*_0_; *β*_1_ and *β*_2_ are fixed effects corresponding to an intercept, the effect of age and gender, respectively. The family structure is modeled through $\boldsymbol{u}\mid {\sigma}_g^2\sim N\left(\mathbf{0},2{\sigma}_g^2\boldsymbol{K}\right)$, where ${\sigma}_g^2$ is the additive genetic variance; ***K*** is the kinship matrix; and ***u*** is the vector of individual breeding values. Because breeding values should be quite stable pre- and posttreatment, we can use the correlation between pre- and posttreatment breeding values to assess possible sample swaps.

## Results

### Probe type

**Fig. 1:**
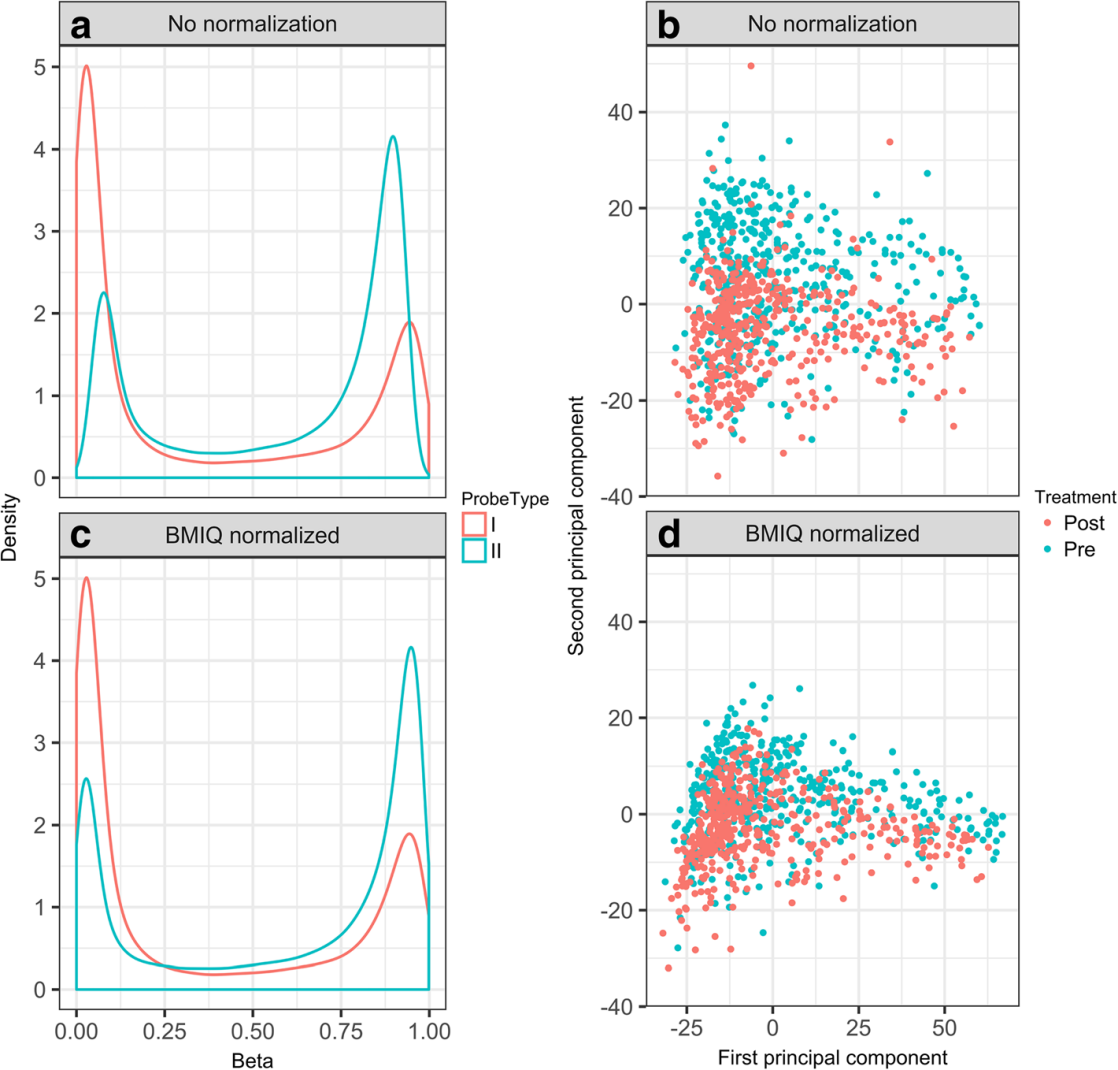
Before (**a**, **b**) and after (**c**, **d**) BMIQ normalization. **a** Distribution of methylation values genome wide for 1 randomly chosen individual before probe-type normalization with BMIQ; **b** PCA plots before probe-type normalization with BMIQ; **c** Distribution of methylation values genome wide for the individual in (**a**) after probe-type normalization with BMIQ; and **d** PCA plots after probe-type normalization with BMIQ

Figure 1a shows a density plot for one individual for all CpGs by probe type. Type II probes have a smaller dynamic range than type I probes, as is typical for 450K Chip data prior to normalization. Systematic differences pre- to posttreatment are seen when plotting the first two principal components of the DNAm data (Fig. 1b). Comparing DNAm pre- and posttreatment (for QC only by *t* test, which is not strictly valid for family data), we identified approximately 300,000 significant CpGs after Bonferroni correction (family-wise error rate = 0.05). Clearly the data, as delivered to the GAW20 participants, has some remaining normalization problems with respect to both probe type and time point.

We performed probe-type normalization with BMIQ. Figure 1c shows the density plot post-BMIQ normalization for the same sample as in Fig. 1a. While this aligned the distributions of the Type I and Type II probes, the PCA plot still shows systematic differences between time points (Fig. 1d). When repeating the QC *t* test, we still found an unrealistically high number (~ 240,000) of differentially methylated CpGs.

### Sample swaps

Without technical covariates such as bisulfite conversion batch or sample position on the 450K Chip (12 subarrays per slide), it is difficult to correct explicitly for residual pre- and posttreatment differences. However, it is possible to use the repeated measures and kinship structure to look for sample swaps and other systematic differences.

**Fig. 2:**
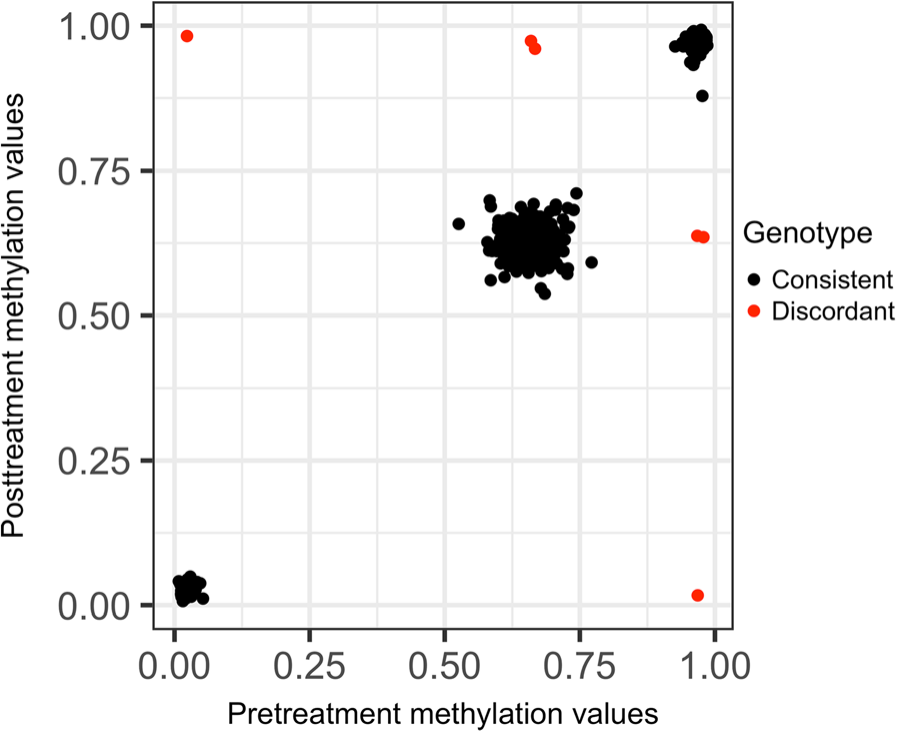
SNPs located in CpGs. Methylation value pre- versus posttreatment for 1 CpG (cg25649515) with the 3 clusters on the diagonal representing the different underlying SNP genotypes at rs552757. Individuals that switch inferred genotype between the 2 measurements are colored red

**Fig. 3:**
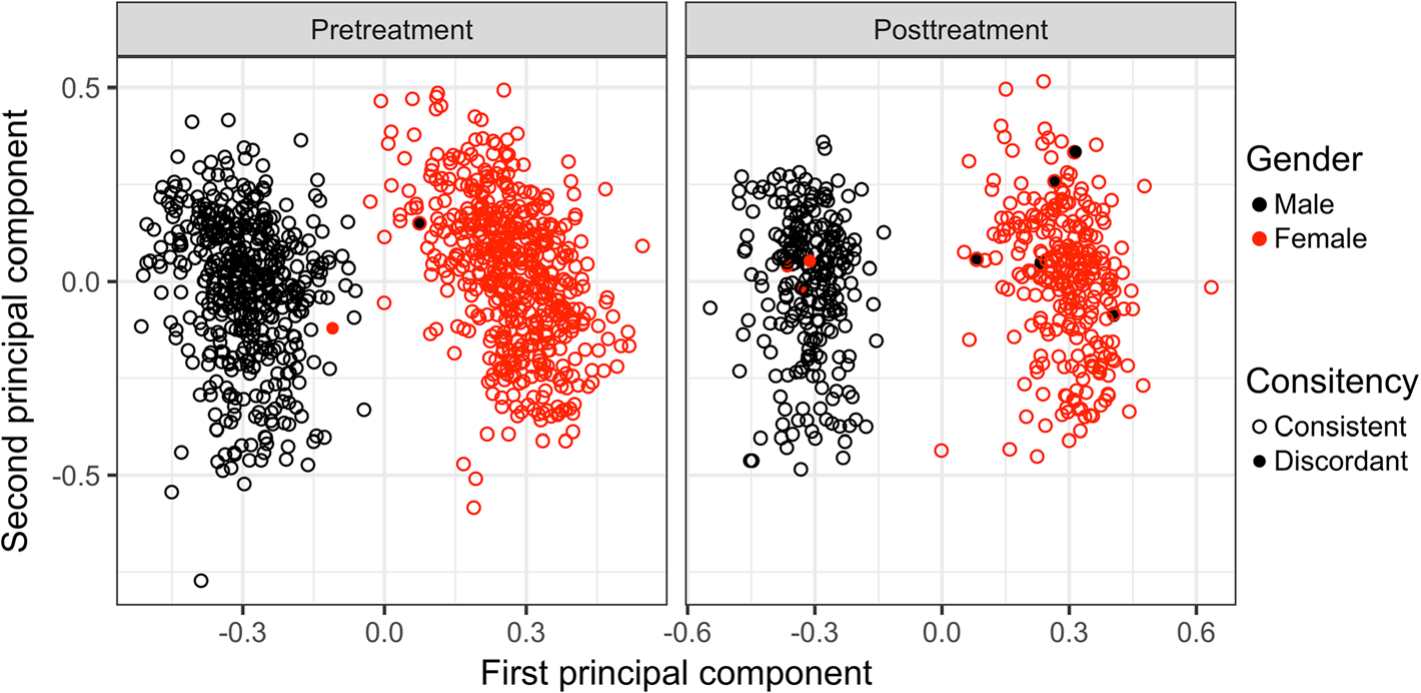
Autosomal CpG-inferred gender. Principal component analysis of 50 gender-associated autosomal CpG sites pre- and posttreatment. The coloring is based on the reported gender and filled points represent “misclassified” individuals

**Fig. 4:**
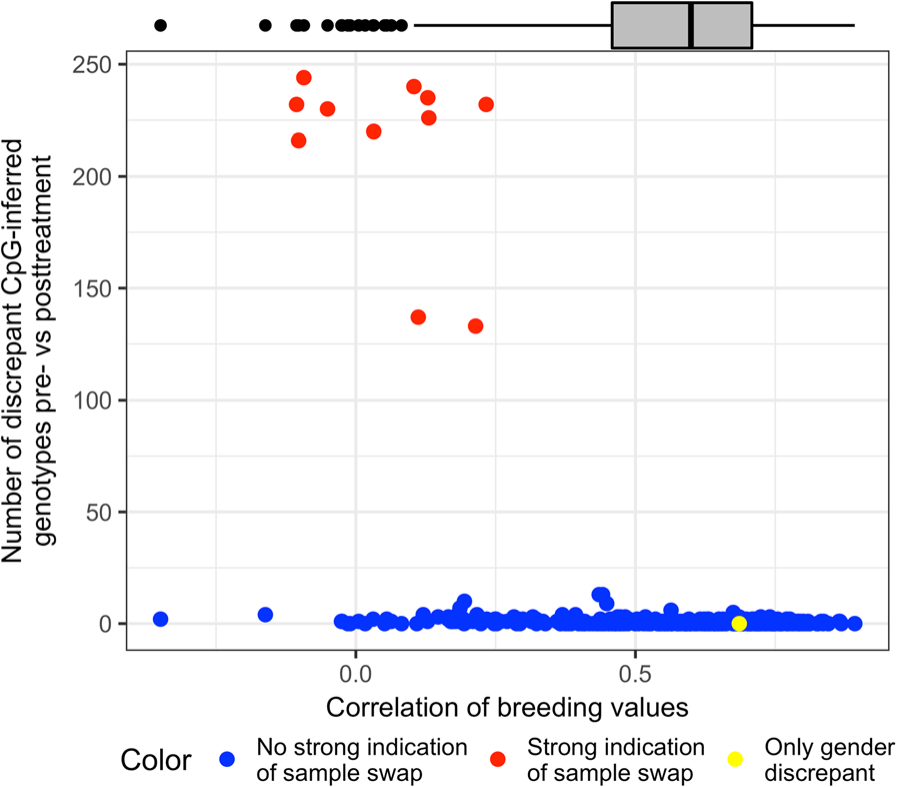
Number of discrepancies for CpG-inferred genotype against the correlation of breeding values. The *y*-axis shows the number of differences between pre- and posttreatment methylation that exceeded 0.25 for 597 SNP-associated CpGs. The *x*-axis shows the estimated correlation of the individual breeding values between pre- and posttreatment methylation across 1000 randomly chosen CpGs. Each individual corresponds to 1 point. The yellow color indicates 1 person whose reported gender is discrepant from its CpG-inferred gender both pre- and posttreatment. A boxplot of the correlation of breeding values for all individuals is also displayed

1. *SNPs located in CpGs:* In Fig. 2, we plot pre- versus posttreatment DNAm for 444 individuals for CpG cg25649515, where SNP rs552757 is located at the CpG. Three clusters corresponding to the AA, AG, and GG genotypes are clearly seen. We also see some individuals (in red) that appear to switch genotype pre- to post- treatment. Using 597 SNP-containing CpGs, we identify 11 individuals who switch the inferred genotype at > 100 loci. For these 11 individuals, we compared CpG-inferred genotype to actual SNP genotype where available, which makes it is easy to visually detect heterozygote to homozygote mismatches. The actual genotype was in agreement with the pretreatment CpG-inferred genotype for 9 individuals and with the posttreatment CpG-inferred genotype for 2 individuals.
2. *CpGs associated with gender:* Because the GAW20 data had only autosomal DNAm available, it was not possible to directly check reported gender against sex chromosomes; instead, we used PCA for the top 50 autosomal CpGs associated with gender in Inoshita et al. [13]. Indeed, 2 gender clusters are clearly visible when the samples are colored by reported gender (Fig. 3). For pretreatment, there are 2 individuals with potentially discordant reported gender and CpG-inferred gender; for posttreatment, there are 8 such individuals (including 1 who was also discordant pretreatment). Six of the 8 individuals with discordant gender posttreatment are among the 11 individuals with SNP-genotype discrepancies; 1 individual was discordant for both time points. We thus identified an additional 3 problematic individuals with this QC step.
3. *Individual breeding values:* We estimated individual breeding values pre- and posttreatment with a Bayesian linear mixed model. We then calculated the correlation between the 2 vectors of breeding values for each individual. Figure 4 shows the number of discrepancies between CpG-inferred genotypes pre- versus posttreatment against the correlation of breeding values. All 11 individuals with more than 100 genotype discrepancies also have low correlation of breeding values (< 0.25). Of the 3 additional gender-discordant individuals, only 1 had both pre- and posttreatment methylation available. This individual’s reported gender was discordant with its CpG-inferred gender at both time points, had a relatively high breeding value correlation and no discrepancies between SNP genotype and CpG-inferred genotype. This individual is likely not a sample swap but rather a misreported gender.

Note that we can estimate the expected number of CpGs with genotype discrepancies in the presence of a sample swap, given that some of the swapped samples might by chance have the same genotype. For a rough estimate (assuming Hardy-Weinberg equilibrium), if all CpGs were associated with SNPs with a minor allele frequency of 0.25, we would expect to be able to identify the sample swap in 53.9% of all CpGs.

## Discussion

We demonstrate several ways to perform QC of DNAm data in the absence of raw data files by using information on pedigree structure, repeated measurements, and external data such as gender. We find that the DNAm data shows systematic differences (a) by probe type and (b) by time point (pre- vs posttreatment). After performing BMIQ normalization for probe type, we demonstrate how SNP genotypes, repeated measures, family structure and external information on gender-associated CpGs can enhance the QC process. We use this additional information to identify up to 13 sample swaps, 11 of which occur posttreatment. We also identify 1 additional individual with a likely misreported gender. The systematic pre- vs posttreatment difference improved after BMIQ normalization, but was not completely resolved.

Because a genome-wide treatment effect on DNAm is unlikely [10], it is possible that pre- and posttreatment methylation samples were run in separate bisulfite conversion batches or run on separate chips, causing treatment to be confounded with batch. This makes any inference of treatment effect on DNAm difficult. How to correct for batch effect when confounded with treatment is not obvious, and is particularly challenging, because the change in methylation from pre- to posttreatment tends to be larger for CpGs with either low or high methylation compared to CpGs with approximately 50% methylation (data not shown), which means that a simple correction for a global mean is not appropriate.

We detect 13 sample swaps using unconventional methods. The 450K Chip is designed with 65 control probes that normally can be used for such purposes, but these were not provided in the GAW20 data. Consequently, we used SNP-containing CpGs as a proxy and looked for CpG-inferred genotype swaps pre- and posttreatment, and compared the inferred genotypes to the actual SNP genotypes where available. The 450K Chip has probes on the sex chromosomes but these were not provided in the GAW20 data. Using PCA of 50 autosomal CpGs previously associated with gender, we identified gender-specific clusters and identified samples whose reported gender was discordant from their PCA-cluster gender. We then used the family structure to estimate individual breeding values for DNAm. Previously identified likely sample swaps were confirmed by low correlation of the individual breeding values across time points. In addition, negative or very low correlation of breeding values between time points could indicate low-quality DNAm at one of the time points.

We identify more likely sample swaps posttreatment compared to pretreatment, which would weaken any relationship between DNAm measured posttreatment and family structure. This is consistent with the markedly lower reported DNAm heritability seen posttreatment compared to pretreatment [14].

## Conclusions

This work highlights the importance of normalization and QC of the GAW20 DNAm data and demonstrates several ways to perform QC of 450K Chip data in the absence of raw data files.

## Abbreviations

450K Chip: Illumina Infinium Human Methylation 450K BeadChip
BMIQ: Beta-mixture quantile normalization
CpG: Cytosine-phosphate-guanine
DNAm: DNA methylation
GAW20: Genetic Analysis Workshop 20
GOLDN: Genetics of Lipid Lowering Drugs and Diet Network
INLA: Integrated nested Laplace approximation
PCA: Principal component analysis
QC: Quality control
SNP: Single nucleotide polymorphism
SWAN: Subset-quantile within array normalization

## References

[1] Bibikova M, Barnes B, Tsan C, Ho V, Klotzle B, Le JM, Delano D, Zhang L, Schroth GP, Gunderson KL (2011). High density DNA methylation array with single CpG site resolution. Genomics.

[2] Aryee MJ, Jaffe AE, Corrada-Bravo H, Ladd-Acosta C, Feinberg AP, Hansen KD, Irizarry RA (2014). Minfi: a flexible and comprehensive Bioconductor package for the analysis of Infinium DNA methylation microarrays. Bioinformatics.

[3] Morris TJ, Butcher LM, Feber A, Teschendorff AE, Chakravarthy AR, Wojdacz TK, Beck S (2014). ChAMP: 450k chip analysis methylation pipeline. Bioinformatics.

[4] Assenov Y, Muller F, Lutsik P, Walter J, Lengauer T, Bock C (2014). Comprehensive analysis of DNA methylation data with RnBeads. Nat Methods.

[5] Davis S, Du P, Bilke S, Triche T Jr, Bootwalla M. Methylumi: handle Illumina methylation data. R package version. 2017;2(0).

[6] Jones PA (2012). Functions of DNA methylation: islands, start sites, gene bodies and beyond. Nat Rev Genet.

[7] Maksimovic J, Gordon L, Oshlack A (2012). SWAN: subset-quantile within array normalization for Illumina Infinium HumanMethylation450 BeadChips. Genome Biol.

[8] Fortin JP, Labbe A, Lemire M, Zanke BW, Hudson TJ, Fertig EJ, Greenwood CM, Hansen KD (2014). Functional normalization of 450k methylation array data improves replication in large cancer studies. Genome Biol.

[9] Teschendorff AE, Marabita F, Lechner M, Bartlett T, Tegner J, Gomez-Cabrero D, Beck S (2013). A beta-mixture quantile normalization method for correcting probe design bias in Illumina Infinium 450k DNA methylation data. Bioinformatics.

[10] Das M, Irvin MR, Sha J, Aslibekyan S, Hidalgo B, Perry RT, Zhi D, Tiwari HK, Absher D, Ordovas JM (2015). Lipid changes due to fenofibrate treatment are not associated with changes in DNA methylation patterns in the GOLDN study. Front Genet.

[11] Aslibekyan S, Almasy L, Province MA, Absher DM, Arnett DK. Data for GAW20: genome-wide DNA Sequence variation and epigenome-wide DNA methylation before and after fenofibrate treatment in a family study of metabolic phenotypes. BMC Proc. 2018;12(Suppl 9). 10.1186/s12919-018-0114-0. 10.1186/s12919-018-0114-0 PMC6157153 30275886

[12] Lai CQ, Arnett DK, Corella D, Straka RJ, Tsai MY, Peacock JM, Adiconis X, Parnell LD, Hixson JE, Province MA (2007). Fenofibrate effect on triglyceride and postprandial response of apolipoprotein A5 variants: the GOLDN study. Arterioscler Thromb Vasc Biol.

[13] Inoshita M, Numata S, Tajima A, Kinoshita M, Umehara H, Yamamori H, Hashimoto R, Imoto I, Ohmori T (2015). Sex differences of leukocytes DNA methylation adjusted for estimated cellular proportions. Biol Sex Differ.

[14] Nustad H, Page CM, Reiner AH, Zucknick M, LeBlanc M. A Bayesian mixed modeling approach for estimating heritability. BMC Proc. 2018;12(Suppl 9). 10.1186/s12919-018-0131-z. 10.1186/s12919-018-0131-z PMC6157283 30275883

[15] Rue H, Martino S, Chopin N (2009). Approximate Bayesian inference for latent Gaussian models by using integrated nested Laplace approximations. J R Stat Soc Series B Stat Methodol.

[16] The R-INLA project. http://www.r-inla.org. Accessed 3 May 2017.

